# Bis(4,4′-methyl­enedicyclo­hexyl­aminium) μ-benzene-1,4-dicarboxyl­ato-bis­[tri­chlorido­zinc(II)] tetra­hydrate

**DOI:** 10.1107/S1600536808033011

**Published:** 2008-10-15

**Authors:** Chen-Yen Hsu, Chun-Wei Yeh, Chi-Phi Wu, Chia-Her Lin, Jhy-Der Chen

**Affiliations:** a Department of Chemistry, Chung-Yuan Christian University, Chung-Li 320, Taiwan

## Abstract

The title compound, (C_13_H_28_N_2_)_2_[Zn_2_(C_8_H_4_O_4_)Cl_6_]·4H_2_O, was prepared by the reaction of ZnCl_2_·6H_2_O, benzene-1,4-dicarboxylic acid and 4,4′-diamino­dicyclo­hexyl­methane in methanol. The [Zn_2_Cl_6_(C_8_H_4_O_4_)]^4−^ anions lie on centres of inversion and comprise two ZnCl_3_ groups bridged by benzene-1,4-dicarboxyl­ate. In addition to N—H⋯Cl and N—H⋯O hydrogen bonds between the cations and anions, solvent water mol­ecules form O—H⋯O and O—H⋯Cl hydrogen bonds to give a three-dimensional network.

## Related literature

For related structures, see: Clausen *et al.* (2005 ▶); Thirumurugan & Rao (2005 ▶); Li *et al.* (1998 ▶, 1999 ▶).
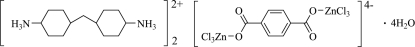


## Experimental

### 

#### Crystal data

(C_13_H_28_N_2_)_2_[Zn_2_(C_8_H_4_O_4_)Cl_6_]·4H_2_O
*M* *_r_* = 1004.36
Monoclinic, 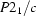

*a* = 14.264 (3) Å
*b* = 14.202 (2) Å
*c* = 11.712 (2) Å
β = 100.498 (16)°
*V* = 2333.0 (7) Å^3^
*Z* = 2
Mo *K*α radiation
μ = 1.42 mm^−1^
*T* = 295 (2) K
0.70 × 0.40 × 0.10 mm

#### Data collection

Bruker *P*4 diffractometer
Absorption correction: ψ scan (*XSCANS*; Siemens, 1995 ▶) *T*_min_ = 0.694, *T*_max_ = 0.868
5093 measured reflections
4071 independent reflections
3405 reflections with *I* > 2σ(*I*)
*R*_int_ = 0.027
3 standard reflections every 97 reflections intensity decay: none

#### Refinement

*R*[*F* ^2^ > 2σ(*F* ^2^)] = 0.057
*wR*(*F* ^2^) = 0.132
*S* = 1.05
4071 reflections
244 parameters
2 restraints
H-atom parameters constrained
Δρ_max_ = 1.10 e Å^−3^
Δρ_min_ = −0.83 e Å^−3^

### 

Data collection: *XSCANS* (Siemens, 1995 ▶); cell refinement: *XSCANS*; data reduction: *SHELXTL* (Sheldrick, 2008 ▶); program(s) used to solve structure: *SHELXS97* (Sheldrick, 2008 ▶); program(s) used to refine structure: *SHELXL97* (Sheldrick, 2008 ▶); molecular graphics: *SHELXTL*; software used to prepare material for publication: *SHELXTL*.

## Supplementary Material

Crystal structure: contains datablocks I, global. DOI: 10.1107/S1600536808033011/bi2304sup1.cif

Structure factors: contains datablocks I. DOI: 10.1107/S1600536808033011/bi2304Isup2.hkl

Additional supplementary materials:  crystallographic information; 3D view; checkCIF report

## Figures and Tables

**Table 1:** Hydrogen-bond geometry (Å, °)

*D*—H⋯*A*	*D*—H	H⋯*A*	*D*⋯*A*	*D*—H⋯*A*
N1—H1*A*⋯Cl3	0.89	2.41	3.252 (4)	159
N1—H1*B*⋯Cl2^i^	0.89	2.51	3.290 (4)	147
N1—H1*C*⋯O3^i^	0.89	1.94	2.828 (5)	178
N2—H2*A*⋯Cl1^ii^	0.89	2.95	3.725 (5)	146
N2—H2*A*⋯Cl2^ii^	0.89	2.67	3.321 (4)	131
N2—H2*B*⋯O2^iii^	0.89	2.06	2.928 (4)	166
N2—H2*C*⋯O4^iv^	0.89	1.95	2.813 (4)	164
O3—H3*B*⋯O2	1.00	1.81	2.798 (4)	167.8
O3—H3*C*⋯Cl1^v^	1.06	2.24	3.262 (4)	160.3
O4—H4*B*⋯Cl3	0.98	2.21	3.172 (4)	164.9
O4—H4*C*⋯Cl2^vi^	0.94	2.38	3.258 (3)	154.9

Symmetry codes: (i) 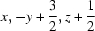; (ii) 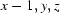; (iii) 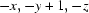; (iv) 
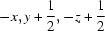
; (v) 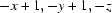; (vi) 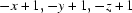.

**Fig. 1.**
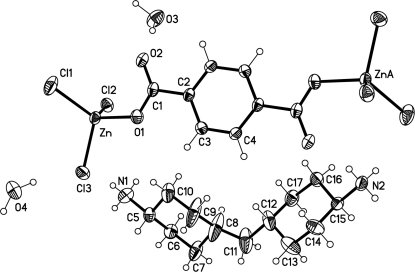
Molecular structure of the title compound with displacement ellipsoids drawn at the 30% probability level for non-H atoms. The [Zn2Cl6(C8H4O4)]4- anion lies on a centre of inversion. Symmetry code: -x, 1 - y, -z.

## supplementary crystallographic information

### Comment

The dianion of benzene-1,4-dicarboxylic acid is an important linker to bridge metal atoms which show significant chemical and physical properties (Clausen *et al.*, 2005; Thirumurugan & Rao, 2005; Li *et al.*, 1998, 1999). Since the anions contain four O atoms which are good hydrogen-bond acceptors, co-crystallization with organic cations would be expected to result in extensive hydrogen-bond networks. The title compound (Fig. 1) contains N—H···Cl and N—H···O hydrogen bonds between the cations and the anions, as well as O—H···O and O—H···Cl interactions formed by the lattice water molecules.

### Experimental

ZnCl_2_.6H_2_O (0.49 g, 2.00 mmol) was added to a solution of 4,4'-diaminodicyclohexylmethane (0.21 g,1.00 mmol) and benzene-1,4-dicarboxylic acid (0.17 g, 1.00 mmol) in 30 ml MeOH. The mixture was refluxed for 24 h to yield a colorless solution with some white solid. The solution was filtered and then diethyl ether was added to induce precipitation. The precipitate was filtered and washed by ether (3 × 10 ml), then dried under reduced pressure to give a white powder. Colourless crystals were obtained by slow diffusion of ether into a methanol solution of the white powder over several weeks.

### Refinement

H atoms bound to C and N atoms were placed in idealized positions and constrained to ride on their parent atoms, with C—H = 0.93–0.98 Å and N—H = 0.89 Å, and with *U*_iso_(H) = 1.2 or 1.5*U*_eq_(C/N). The H atoms of the water molecules were located in difference Fourier maps, then constrained to ride on their parent O atom with *U*_iso_(H) = 1.5*U*_eq_(O). The C8—C11 and C11—C12 bond distances were restrained to be identical with a standard uncertainty of 0.02 Å.

### Crystal data

**Table:**

(C_13_H_28_N_2_)_2_[Zn_2_(C_8_H_4_O_4_)Cl_6_]·4H_2_O	*F*(000) = 1052
*M**_r_* = 1004.36	*D*_x_ = 1.430 Mg m^−^^3^
Monoclinic, *P*2_1_/*c*	Mo *K*α radiation, λ = 0.71073 Å
Hall symbol: -P 2ybc	Cell parameters from 33 reflections
*a* = 14.264 (3) Å	θ = 5.7–12.5°
*b* = 14.202 (2) Å	µ = 1.42 mm^−^^1^
*c* = 11.712 (2) Å	*T* = 295 K
β = 100.498 (16)°	Plate, colourless
*V* = 2333.0 (7) Å^3^	0.70 × 0.40 × 0.10 mm
*Z* = 2	

### Data collection

**Table:**

Bruker P4 diffractometer	3405 reflections with *I* > 2σ(*I*)
Radiation source: fine-focus sealed tube	*R*_int_ = 0.027
graphite	θ_max_ = 25.0°, θ_min_ = 2.0°
ω scans	*h* = −16→16
Absorption correction: ψ scan (XSCANS; Siemens, 1995)	*k* = −1→16
*T*_min_ = 0.694, *T*_max_ = 0.868	*l* = −1→13
5093 measured reflections	3 standard reflections every 97 reflections
4071 independent reflections	intensity decay: none

### Refinement

**Table:**

Refinement on *F*^2^	Primary atom site location: structure-invariant direct methods
Least-squares matrix: full	Secondary atom site location: difference Fourier map
*R*[*F*^2^ > 2σ(*F*^2^)] = 0.057	Hydrogen site location: inferred from neighbouring sites
*wR*(*F*^2^) = 0.132	H-atom parameters constrained
*S* = 1.05	*w* = 1/[σ^2^(*F*_o_^2^) + (0.0344*P*)^2^ + 8.1458*P*] where *P* = (*F*_o_^2^ + 2*F*_c_^2^)/3
4071 reflections	(Δ/σ)_max_ < 0.001
244 parameters	Δρ_max_ = 1.10 e Å^−^^3^
2 restraints	Δρ_min_ = −0.83 e Å^−^^3^

### Special details

**Table:**

Geometry. All e.s.d.'s (except the e.s.d. in the dihedral angle between two l.s. planes) are estimated using the full covariance matrix. The cell e.s.d.'s are taken into account individually in the estimation of e.s.d.'s in distances, angles and torsion angles; correlations between e.s.d.'s in cell parameters are only used when they are defined by crystal symmetry. An approximate (isotropic) treatment of cell e.s.d.'s is used for estimating e.s.d.'s involving l.s. planes.
Refinement. Refinement of *F*^2^ against ALL reflections. The weighted *R*-factor *wR* and goodness of fit *S* are based on *F*^2^, conventional *R*-factors *R* are based on *F*, with *F* set to zero for negative *F*^2^. The threshold expression of *F*^2^ > σ(*F*^2^) is used only for calculating *R*-factors(gt) *etc*. and is not relevant to the choice of reflections for refinement. *R*-factors based on *F*^2^ are statistically about twice as large as those based on *F*, and *R*- factors based on ALL data will be even larger.

### Fractional atomic coordinates and isotropic or equivalent isotropic displacement parameters (Å^2^)

**Table:**

	*x*	*y*	*z*	*U*_iso_*/*U*_eq_	
Zn	0.37495 (3)	0.50908 (4)	0.23918 (5)	0.04083 (17)	
Cl1	0.48147 (11)	0.41661 (13)	0.17455 (16)	0.0807 (5)	
Cl2	0.41895 (9)	0.66231 (9)	0.22022 (11)	0.0522 (3)	
Cl3	0.37246 (11)	0.48512 (10)	0.42863 (12)	0.0611 (4)	
O1	0.2401 (2)	0.4944 (3)	0.1703 (3)	0.0528 (9)	
O2	0.2553 (2)	0.4968 (3)	−0.0161 (3)	0.0515 (8)	
O3	0.3060 (3)	0.6499 (3)	−0.1424 (4)	0.0829 (14)	
H3B	0.2830	0.5919	−0.1077	0.124*	
H3C	0.3735	0.6415	−0.1668	0.124*	
O4	0.4724 (3)	0.2921 (3)	0.5161 (3)	0.0663 (11)	
H4B	0.4531	0.3555	0.4871	0.099*	
H4C	0.4917	0.2894	0.5972	0.099*	
N1	0.3214 (3)	0.6947 (3)	0.5109 (4)	0.0624 (12)	
H1A	0.3470	0.6458	0.4802	0.094*	
H1B	0.3575	0.7097	0.5787	0.094*	
H1C	0.3179	0.7437	0.4629	0.094*	
N2	−0.3906 (3)	0.6298 (3)	0.0987 (4)	0.0495 (10)	
H2A	−0.4399	0.6000	0.1193	0.074*	
H2B	−0.3577	0.5897	0.0629	0.074*	
H2C	−0.4119	0.6769	0.0508	0.074*	
C1	0.2069 (3)	0.4959 (3)	0.0619 (4)	0.0392 (10)	
C2	0.0992 (3)	0.4974 (3)	0.0298 (4)	0.0365 (9)	
C3	0.0434 (3)	0.4915 (4)	0.1146 (4)	0.0447 (11)	
H3A	0.0724	0.4857	0.1922	0.054*	
C4	−0.0549 (3)	0.4940 (3)	0.0853 (4)	0.0425 (11)	
H4A	−0.0915	0.4900	0.1432	0.051*	
C5	0.2238 (3)	0.6694 (4)	0.5292 (5)	0.0494 (12)	
H5A	0.2286	0.6161	0.5831	0.059*	
C6	0.1788 (4)	0.7515 (4)	0.5812 (5)	0.0599 (14)	
H6A	0.2171	0.7677	0.6558	0.072*	
H6B	0.1766	0.8058	0.5306	0.072*	
C7	0.0792 (4)	0.7268 (6)	0.5970 (6)	0.087 (2)	
H7A	0.0504	0.7796	0.6301	0.104*	
H7B	0.0806	0.6733	0.6487	0.104*	
C8	0.0217 (4)	0.7026 (8)	0.4758 (9)	0.153 (5)	
H8A	0.0323	0.7544	0.4244	0.183*	
C9	0.0658 (4)	0.6144 (7)	0.4308 (9)	0.144 (5)	
H9A	0.0680	0.5633	0.4862	0.173*	
H9B	0.0278	0.5943	0.3576	0.173*	
C10	0.1653 (4)	0.6397 (5)	0.4146 (6)	0.084 (2)	
H10A	0.1949	0.5858	0.3845	0.101*	
H10B	0.1626	0.6907	0.3590	0.101*	
C11	−0.0777 (4)	0.6972 (7)	0.4707 (7)	0.129 (4)	
H11A	−0.0985	0.7584	0.4932	0.154*	
H11B	−0.0886	0.6529	0.5299	0.154*	
C12	−0.1428 (5)	0.6698 (5)	0.3598 (5)	0.082 (2)	
H12A	−0.1106	0.6224	0.3200	0.099*	
C13	−0.2310 (6)	0.6253 (5)	0.3937 (6)	0.086 (2)	
H13A	−0.2119	0.5736	0.4470	0.104*	
H13B	−0.2637	0.6716	0.4331	0.104*	
C14	−0.2979 (5)	0.5892 (4)	0.2885 (5)	0.0648 (16)	
H14A	−0.2665	0.5405	0.2512	0.078*	
H14B	−0.3538	0.5619	0.3119	0.078*	
C15	−0.3274 (3)	0.6681 (3)	0.2049 (4)	0.0448 (11)	
H15A	−0.3640	0.7138	0.2416	0.054*	
C16	−0.2440 (4)	0.7187 (4)	0.1693 (5)	0.0568 (14)	
H16A	−0.2669	0.7726	0.1214	0.068*	
H16B	−0.2118	0.6767	0.1237	0.068*	
C17	−0.1739 (4)	0.7515 (4)	0.2759 (5)	0.0657 (16)	
H17A	−0.2035	0.8001	0.3155	0.079*	
H17B	−0.1182	0.7786	0.2516	0.079*	

### Atomic displacement parameters (Å^2^)

**Table:**

	*U*^11^	*U*^22^	*U*^33^	*U*^12^	*U*^13^	*U*^23^
Zn	0.0279 (3)	0.0487 (3)	0.0448 (3)	0.0002 (2)	0.0038 (2)	−0.0039 (2)
Cl1	0.0513 (8)	0.0921 (12)	0.0964 (12)	0.0202 (8)	0.0077 (8)	−0.0412 (10)
Cl2	0.0443 (6)	0.0524 (7)	0.0572 (7)	−0.0055 (5)	0.0023 (5)	0.0085 (6)
Cl3	0.0727 (9)	0.0612 (8)	0.0493 (7)	0.0159 (7)	0.0108 (6)	0.0109 (6)
O1	0.0318 (16)	0.074 (2)	0.050 (2)	−0.0063 (17)	−0.0002 (14)	−0.0007 (18)
O2	0.0348 (17)	0.063 (2)	0.060 (2)	0.0050 (16)	0.0154 (15)	0.0004 (17)
O3	0.086 (3)	0.063 (3)	0.112 (4)	0.007 (2)	0.051 (3)	0.012 (2)
O4	0.074 (3)	0.052 (2)	0.065 (2)	0.0020 (19)	−0.010 (2)	0.0026 (18)
N1	0.059 (3)	0.057 (3)	0.074 (3)	−0.014 (2)	0.020 (2)	−0.018 (2)
N2	0.034 (2)	0.059 (3)	0.056 (2)	−0.0009 (19)	0.0076 (18)	−0.004 (2)
C1	0.032 (2)	0.030 (2)	0.055 (3)	0.0004 (18)	0.006 (2)	−0.005 (2)
C2	0.028 (2)	0.035 (2)	0.046 (2)	−0.0022 (18)	0.0072 (18)	−0.0064 (19)
C3	0.036 (2)	0.059 (3)	0.038 (2)	−0.001 (2)	0.0023 (19)	−0.002 (2)
C4	0.035 (2)	0.054 (3)	0.040 (2)	0.000 (2)	0.0110 (19)	−0.003 (2)
C5	0.041 (3)	0.044 (3)	0.060 (3)	−0.001 (2)	0.001 (2)	−0.006 (2)
C6	0.057 (3)	0.064 (3)	0.056 (3)	0.001 (3)	0.003 (3)	−0.022 (3)
C7	0.042 (3)	0.116 (6)	0.097 (5)	0.002 (3)	−0.001 (3)	−0.063 (5)
C8	0.044 (4)	0.219 (11)	0.181 (9)	0.025 (5)	−0.015 (5)	−0.162 (9)
C9	0.038 (3)	0.181 (9)	0.198 (10)	0.017 (5)	−0.018 (5)	−0.149 (8)
C10	0.065 (4)	0.097 (5)	0.081 (4)	0.030 (4)	−0.013 (3)	−0.049 (4)
C11	0.084 (5)	0.167 (9)	0.113 (6)	0.057 (6)	−0.042 (5)	−0.090 (6)
C12	0.077 (4)	0.090 (5)	0.066 (4)	0.044 (4)	−0.024 (3)	−0.038 (4)
C13	0.120 (6)	0.074 (4)	0.054 (4)	−0.001 (4)	−0.013 (4)	−0.001 (3)
C14	0.089 (4)	0.052 (3)	0.049 (3)	−0.010 (3)	0.003 (3)	−0.001 (3)
C15	0.040 (2)	0.048 (3)	0.047 (3)	0.000 (2)	0.011 (2)	−0.004 (2)
C16	0.046 (3)	0.063 (3)	0.059 (3)	−0.011 (3)	0.005 (2)	0.012 (3)
C17	0.046 (3)	0.071 (4)	0.079 (4)	−0.011 (3)	0.009 (3)	−0.011 (3)

### Geometric parameters (Å, °)

**Table:**

Zn—O1	1.956 (3)	C7—C8	1.543 (9)
Zn—Cl1	2.2418 (15)	C7—H7A	0.97
Zn—Cl3	2.2514 (15)	C7—H7B	0.97
Zn—Cl2	2.2869 (14)	C8—C11	1.410 (7)
O1—C1	1.272 (6)	C8—C9	1.537 (10)
O2—C1	1.243 (6)	C8—H8A	0.98
O3—H3B	1.00	C9—C10	1.510 (11)
O3—H3C	1.06	C9—H9A	0.97
O4—H4B	0.98	C9—H9B	0.97
O4—H4C	0.94	C10—H10A	0.97
N1—C5	1.491 (6)	C10—H10B	0.97
N1—H1A	0.89	C11—C12	1.503 (6)
N1—H1B	0.89	C11—H11A	0.97
N1—H1C	0.89	C11—H11B	0.97
N2—C15	1.500 (6)	C12—C13	1.524 (11)
N2—H2A	0.89	C12—C17	1.534 (9)
N2—H2B	0.89	C12—H12A	0.98
N2—H2C	0.89	C13—C14	1.503 (8)
C1—C2	1.513 (6)	C13—H13A	0.97
C2—C3	1.385 (6)	C13—H13B	0.97
C2—C4^i^	1.386 (6)	C14—C15	1.497 (7)
C3—C4	1.382 (6)	C14—H14A	0.97
C3—H3A	0.93	C14—H14B	0.97
C4—C2^i^	1.386 (6)	C15—C16	1.512 (7)
C4—H4A	0.93	C15—H15A	0.98
C5—C10	1.505 (7)	C16—C17	1.523 (8)
C5—C6	1.512 (7)	C16—H16A	0.97
C5—H5A	0.98	C16—H16B	0.97
C6—C7	1.506 (8)	C17—H17A	0.97
C6—H6A	0.97	C17—H17B	0.97
C6—H6B	0.97		
			
O1—Zn—Cl1	118.31 (12)	C7—C8—H8A	106.4
O1—Zn—Cl3	101.50 (11)	C10—C9—C8	107.6 (7)
Cl1—Zn—Cl3	112.19 (7)	C10—C9—H9A	110.2
O1—Zn—Cl2	109.14 (12)	C8—C9—H9A	110.2
Cl1—Zn—Cl2	108.05 (7)	C10—C9—H9B	110.2
Cl3—Zn—Cl2	107.06 (6)	C8—C9—H9B	110.2
C1—O1—Zn	124.7 (3)	H9A—C9—H9B	108.5
H3B—O3—H3C	113.5	C5—C10—C9	109.6 (6)
H4B—O4—H4C	113.5	C5—C10—H10A	109.8
C5—N1—H1A	109.5	C9—C10—H10A	109.8
C5—N1—H1B	109.5	C5—C10—H10B	109.8
H1A—N1—H1B	109.5	C9—C10—H10B	109.8
C5—N1—H1C	109.5	H10A—C10—H10B	108.2
H1A—N1—H1C	109.5	C8—C11—C12	120.6 (7)
H1B—N1—H1C	109.5	C8—C11—H11A	107.2
C15—N2—H2A	109.5	C12—C11—H11A	107.2
C15—N2—H2B	109.5	C8—C11—H11B	107.2
H2A—N2—H2B	109.5	C12—C11—H11B	107.2
C15—N2—H2C	109.5	H11A—C11—H11B	106.8
H2A—N2—H2C	109.5	C11—C12—C13	107.0 (6)
H2B—N2—H2C	109.5	C11—C12—C17	114.7 (7)
O2—C1—O1	125.4 (4)	C13—C12—C17	109.0 (5)
O2—C1—C2	119.5 (4)	C11—C12—H12A	108.6
O1—C1—C2	115.2 (4)	C13—C12—H12A	108.6
C3—C2—C4^i^	118.9 (4)	C17—C12—H12A	108.6
C3—C2—C1	120.7 (4)	C14—C13—C12	111.0 (6)
C4^i^—C2—C1	120.4 (4)	C14—C13—H13A	109.4
C4—C3—C2	120.7 (4)	C12—C13—H13A	109.4
C4—C3—H3A	119.7	C14—C13—H13B	109.4
C2—C3—H3A	119.7	C12—C13—H13B	109.4
C3—C4—C2^i^	120.4 (4)	H13A—C13—H13B	108.0
C3—C4—H4A	119.8	C15—C14—C13	110.0 (5)
C2^i^—C4—H4A	119.8	C15—C14—H14A	109.7
N1—C5—C10	108.6 (5)	C13—C14—H14A	109.7
N1—C5—C6	110.4 (4)	C15—C14—H14B	109.7
C10—C5—C6	111.6 (4)	C13—C14—H14B	109.7
N1—C5—H5A	108.7	H14A—C14—H14B	108.2
C10—C5—H5A	108.7	C14—C15—N2	109.0 (4)
C6—C5—H5A	108.7	C14—C15—C16	113.2 (4)
C7—C6—C5	110.4 (5)	N2—C15—C16	109.2 (4)
C7—C6—H6A	109.6	C14—C15—H15A	108.4
C5—C6—H6A	109.6	N2—C15—H15A	108.4
C7—C6—H6B	109.6	C16—C15—H15A	108.4
C5—C6—H6B	109.6	C15—C16—C17	110.6 (5)
H6A—C6—H6B	108.1	C15—C16—H16A	109.5
C6—C7—C8	107.2 (6)	C17—C16—H16A	109.5
C6—C7—H7A	110.3	C15—C16—H16B	109.5
C8—C7—H7A	110.3	C17—C16—H16B	109.5
C6—C7—H7B	110.3	H16A—C16—H16B	108.1
C8—C7—H7B	110.3	C16—C17—C12	111.3 (5)
H7A—C7—H7B	108.5	C16—C17—H17A	109.4
C11—C8—C9	114.4 (7)	C12—C17—H17A	109.4
C11—C8—C7	114.4 (7)	C16—C17—H17B	109.4
C9—C8—C7	108.3 (7)	C12—C17—H17B	109.4
C11—C8—H8A	106.4	H17A—C17—H17B	108.0
C9—C8—H8A	106.4		

Symmetry codes: (i) −*x*, −*y*+1, −*z*.

### Hydrogen-bond geometry (Å, °)

**Table:**

*D*—H···*A*	*D*—H	H···*A*	*D*···*A*	*D*—H···*A*
N1—H1A···Cl3	0.89	2.41	3.252 (4)	159
N1—H1B···Cl2^ii^	0.89	2.51	3.290 (4)	147
N1—H1C···O3^ii^	0.89	1.94	2.828 (5)	178
N2—H2A···Cl1^iii^	0.89	2.95	3.725 (5)	146
N2—H2A···Cl2^iii^	0.89	2.67	3.321 (4)	131
N2—H2B···O2^i^	0.89	2.06	2.928 (4)	166
N2—H2C···O4^iv^	0.89	1.95	2.813 (4)	164
O3—H3B···O2	1.00	1.81	2.798 (4)	167.8
O3—H3C···Cl1^v^	1.06	2.24	3.262 (4)	160.3
O4—H4B···Cl3	0.98	2.21	3.172 (4)	164.9
O4—H4C···Cl2^vi^	0.94	2.38	3.258 (3)	154.9

Symmetry codes: (ii) *x*, −*y*+3/2, *z*+1/2; (iii) *x*−1, *y*, *z*; (i) −*x*, −*y*+1, −*z*; (iv) −*x*, *y*+1/2, −*z*+1/2; (v) −*x*+1, −*y*+1, −*z*; (vi) −*x*+1, −*y*+1, −*z*+1.
